# The Roo*Pf*s study to assess whether improved housing provides additional protection against clinical malaria over current best practice in The Gambia: study protocol for a randomized controlled study and ancillary studies

**DOI:** 10.1186/s13063-016-1400-7

**Published:** 2016-06-03

**Authors:** Margaret Pinder, Lesong Conteh, David Jeffries, Caroline Jones, Jakob Knudsen, Balla Kandeh, Musa Jawara, Elisa Sicuri, Umberto D’Alessandro, Steve W. Lindsay

**Affiliations:** School of Biological and Biomedical Sciences, Durham University, Durham, UK; Medical Research Council’s (MRC) Unit The Gambia, Banjul, The Gambia; Health Economics Group, Department of Infectious Disease Epidemiology, School of Public Health, Imperial College, London, UK; Centre for Tropical Medicine and Global Health, University of Oxford/Kemri-Wellcome Trust Research Programme, Kilifi, Kenya; Schools of Architecture, Design and Conservation (KADK), Copenhagen, Denmark; National Malaria Control Programme, Banjul, The Gambia

**Keywords:** Clinical malaria, Cost-effectiveness, Housing, Household randomized controlled trial, House screening, Insecticide-treated bed net, Long-lasting insecticidal nets, Malaria control

## Abstract

**Background:**

In malaria-endemic areas, residents of modern houses have less malaria than those living in traditional houses. This study will determine if modern housing provides incremental protection against clinical malaria over the current best practice of long-lasting insecticidal nets (LLINs) and prompt treatment in The Gambia, determine the incremental cost-effectiveness of the interventions, and analyze the housing market in The Gambia.

**Methods/design:**

A two-armed, household, cluster-randomized, controlled study will be conducted to assess whether improved housing and LLINs combine to provide better protection against clinical malaria in children than LLINs alone in The Gambia. The unit of randomization will be the household, defined as a house and its occupants. A total of 800 households will be enrolled and will receive LLINs, and 400 will receive improved housing before clinical follow-up. One child aged 6 months to 13 years will be enrolled from each household and followed for clinical malaria using active case detection to estimate malaria incidence for two malaria transmission seasons. Episodes of clinical malaria will be the primary endpoint. Study children will be surveyed at the end of each transmission season to estimate the prevalence of *Plasmodium falciparum* infection, parasite density, and the prevalence of anemia. Exposure to malaria parasites will be assessed using light traps, followed by detection of *Anopheles gambiae* species and sporozoite infection. Ancillary economic and social science studies will undertake a cost-effectiveness analysis and use qualitative and participatory methods to explore the acceptability of the housing modifications and to design strategies for scaling-up housing interventions.

**Discussion:**

The study is the first of its kind to measure the efficacy of housing on reducing clinical malaria, assess the incremental cost-effectiveness of improved housing, and identify mechanisms for scaling up housing interventions. Trial findings will help inform policy makers on improved housing for malaria control in sub-Saharan Africa.

**Trial registration:**

ISRCTN Registry, ISRCTN02622179. Registered on 23 September 2014.

**Electronic supplementary material:**

The online version of this article (doi:10.1186/s13063-016-1400-7) contains supplementary material, which is available to authorized users.

## Background

There have been considerable gains made in malaria control in sub-Saharan Africa, with malaria prevalence dropping by half, and the incidence of clinical disease falling by 40 % from 2000 to 2015 [1]. Yet the disease remains a substantial public health problem, with 188 million cases and 395,000 malaria deaths in 2015 [2]. The reduction in malaria has been achieved largely by the massive scaling up of vector control, with long-lasting insecticidal nets (LLIN) and indoor residual spraying (IRS). However, the future success of these interventions is threatened by the growing problem of insecticide-resistant mosquitoes [3], some of which are resistant to all four classes of insecticide currently available for use in public health [4]. Thus, an urgent need exists to develop supplementary interventions that are not reliant on insecticides. This objective is also advocated by Roll Back Malaria (RBM) and the United Nations Development Program (UNDP, which has stated the need for “making actions outside the health sector essential elements of malaria control” [5]. The need for “good” housing to reduce malaria is echoed throughout this RBM/UNDP document. Recently, the RBM Board [6], noting that continued progress in the fight against malaria is central to the attainment of the Sustainable Development Goals, has committed to implementing the aim through development of a work plan that reaches beyond the traditional health sector to include “environment, climate change, housing, sanitation, agriculture, education, and other sectors invested in the fight against poverty.” Thus, a growing enthusiasm exists for using housing as an intervention against malaria, as illustrated further by the new RBM workstream on malaria and housing (http://archiverbm.rollbackmalaria.org/mechanisms/vcwgWorkstream9.html).

Is housing protective? Improved housing was thought to have contributed to the elimination of malaria in the USA [7, 8] and to its decline in Europe [9]. Narrative reviews suggested that “good housing” is protective in many tropical countries [10, 11]. More recently, a systematic review and meta-analysis of the literature on housing and malaria provided stronger evidence that “good housing” is protective against malaria [12]. Residents of modern homes had 42 % lower odds of malaria infection compared to traditional homes and a 54–65 % lower incidence of clinical malaria. Yet the overall evidence indicates low quality, and only one randomized controlled trial has been conducted of house screening to date. This trial showed that the use of simple untreated screens on the doors and windows and closing the eaves in typical rural African houses reduced the prevalence of anemia in children by 50 % [13]. Nonetheless, the impact of “good” housing on clinical episodes of malaria, the benchmark measurement for assessing malaria interventions, has not been quantified.

The hypothesis supporting housing as being protective against malaria in sub-Saharan Africa is based on the observation that 79–100 % of malaria transmission occurs indoors at night [14]; so preventing the entry of malaria mosquitoes indoors [10, 13] should dramatically reduce malaria risk. Traditional thatched-roofed houses nearly always have open eaves, with a gap between the top of the wall and the roof, through which *Anopheles gambiae*, the principal African malaria vector, enters the house. Metal-roofed houses more frequently have closed eaves, so installing metal-roofs and closing the eaves should reduce the entry of malaria vectors. Because screening the doors and windows also reduces mosquito house entry [13], we hypothesize that a combination of metal roofs, closed eaves, and the installation of screening on the doors and windows should measurably reduce clinical malaria. In addition, to encourage air circulation and help cool the room, while also preventing the entry of mosquitoes into the intervention houses, we propose to install screened windows and doors. Although we consider the interventions safe, the possibility exists that the reduced indoor airflow may increase respiratory disease in children living in screened houses. For this reason, we will also measure respiratory illness in our study cohort.

The long-term effectiveness and potential impact of housing as a malaria intervention will depend on its cost-effectiveness, acceptability to householders, and the feasibility of implementation at scale. Therefore, alongside the trial, we will undertake a cost-effectiveness analysis and use qualitative and participatory methods to explore the acceptability of the housing modifications and to design strategies for scaling up housing interventions. The incremental costs and benefits associated with house improvements, as well as the demand and supply of construction material and skill, are invaluable data to inform policymakers in their decision to adopt the interventions as policy. However, very limited evidence exists on the cost-effectiveness of improved housing on health, and the little information available is focused on interventions implemented in high-income countries [15]. Analysis of the housing sector in sub-Saharan Africa is growing, but most of the studies are concentrated in a few countries and in urban areas [16].

We view improved housing not as a stand-alone-intervention but as one that should be incorporated with established and effective vector-control interventions such as LLINs. Thus, our goal is to determine whether there is any additional benefit in improving rural homes over the current best practice of using LLINs alone for protecting children against clinical malaria, to determine the incremental cost-effectiveness of the interventions, and analyze the housing market in The Gambia.

### Study objectives

#### Clinical

##### Primary objective

The primary clinical objective its to assess whether improved housing (metal roof, closed eaves, improved ventilation, and screened doors and windows) and LLINs provide added protection against clinical malaria in children with the LLINs alone over two malaria transmission seasons of follow-up.

### Secondary objectives

The secondary clinical objectives include the following:

- To determine whether improved housing reduces the rate of parasite infection, parasite density, and anemia in children
- To assess whether improved housing is associated with a rise in respiratory infections

#### Entomological

##### Primary objective

The primary entomological objective is to assess whether improved housing and LLINs reduce vector density inside houses when compared with the LLINs alone.

#### Economic and social science

##### Primary objective

The primary economic and social science objective is to assess the incremental costs, benefits, and cost-effectiveness of these interventions.

### Secondary objective

The secondary economic and social science objectives are as follows:

- To explore the acceptability of the house modifications to the residents
- To analyze the housing market in The Gambia for the identification of economic barriers to scale-up

## Methods/design

### Study area and participant eligibility

The study is situated in the far east of The Gambia, in the Upper River Region (URR), an area of open Sudan savanna. The climate consists of a single rainy season from May to October followed by a long dry season. This defines the highly seasonal malaria transmission, with most malaria episodes experienced during or immediately following the rainy season. This region covers an area of 1995 km^2^ and is bisected by the river into the north and south banks. The population of the region was 182,586 in 2003, the most recent census, with most living in small discrete rural villages in houses made with mud or cement walls and thatched or metal roofs. The study’s field station is based at Basse Santa Su town (UTM coordinates 13.3167 N, -14.2167 W), which is the only sizeable urban area in the region.

Rural villages located on the north and south bank of the Gambia River will be selected. We will discuss the study with village leaders, including women in each potential study village, and proceed if given approval. Community sensitization will be followed by a survey to identify suitable houses.

A total of 800 traditional thatched-roofed houses constructed with mud walls, open eaves, and without ceilings or screening, with two children aged 6 months to 13 years, will be selected. Consent will be sought from the house owners (and residents) for them to join the study by field assistants (Additional file 1).

Once all houses in the intervention arm have been modified, we will seek consent from the parents/carers for a resident child to join the study cohort from both arms of the study (using supplementary materials). After explaining the purpose of the study to the household heads, only children whose parents have given written, informed consent for their child to be included in the study will be enrolled (Additional file 2). The purpose of the study and requirements of the study according to the capability and assent sought will be explained to eligible children > 12 years old.

Each arm of the study will contain 400 houses, and one resident child will be enrolled from each house. The child will be selected randomly from those aged 6 months to 13 years, except if they expect to be away during several month of the transmission season. No distinctions will be made regarding gender or ethnic group. As much as possible, we will encourage the residents of the study houses not to move house during the study.

### Design

**Fig. 1:**
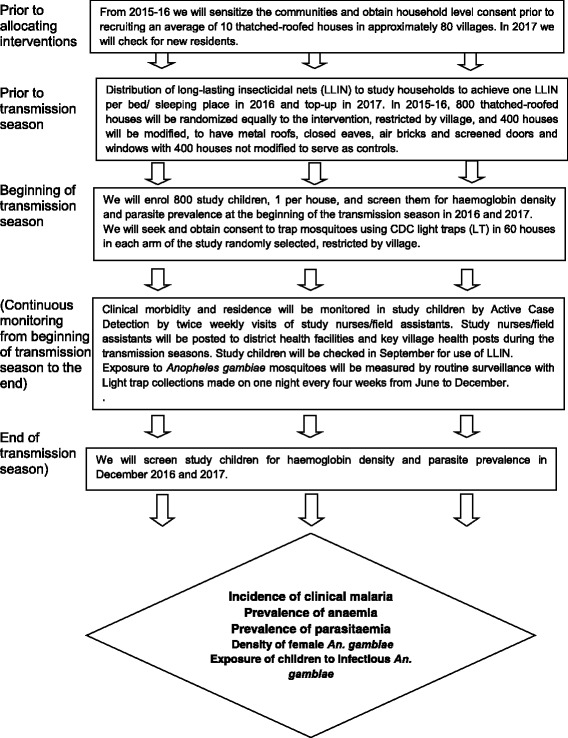
Schematic representation of the trial

The study design is summarized in Fig. 1 and is a two-armed household-clustered randomized controlled study using a generalized, randomized, complete, block design, with the village as the block. At least two control and two intervention houses will be used from each village, but interventions will not be introduced in > 10 % of houses in each village to reduce the chance that mosquitoes prevented from entering an intervention house will enter a control house in greater numbers than normal. Previous studies in The Gambia suggest that this risk is low and that mosquito diversion is unlikely to increase exposure in unprotected homes [17, 18]. A total of 400 houses will be included in each arm of the study, and one resident child will be enrolled from each house. If the resident child enrolled in the study leaves the house for whatever reason, they will be replaced by another child from the same house. The clinical study will take place over 2 years, with clinical follow-up and entomological studies being conducted over two consecutive transmission seasons (June to December); at the end of this period, households in the control group will be provided with a metal roof and screening if they desire it. Children will be enrolled at the start of the first transmission season and will be followed for clinical malaria using active case detection (ACD) to estimate malaria incidence for two malaria transmission seasons. These children will also be surveyed at the beginning and end of each transmission season to estimate the prevalence of *Plasmodium falciparum* infection, parasite density, and the prevalence of anemia. Exposure to malaria parasites will be assessed using light traps in 60 houses in each arm of the study, followed by detection of *An. gambiae* species and sporozoite infection.

Ancillary economic and social science studies will undertake a cost-effectiveness analysis and use qualitative and participatory methods to explore the acceptability of the housing modifications and to design strategies for scaling-up housing interventions. The schedule of enrolment, interventions and assessment is shown in Additional file 3.

### Randomization

Since there is considerable variation in malaria risk between villages [19], houses will be randomized to the study arms by MP, stratified by village using a computer subroutine in a blinded manner so that an equal number of houses are selected in each arm of the study in each village at baseline. Every effort will be made to ensure that the arms of the study are balanced by number and village. Furthermore, if a house leaves the study in year 1, efforts will be made to replace it in year 2. Once the intervention is in place, one child will be selected randomly and recruited from each house. The PI and trial statistician are responsible for the house and child identification, as well as the enrollment and intervention assignment.

A total of 120 houses will be selected for monthly light trap collections of mosquitoes from the 800 houses enrolled, stratified by intervention, village, and geography so that an equal number of houses are selected in each arm of the study in each village, and the subsample is equally spread over the study area.

Stratified randomization by village will reduce the likelihood of chance imbalances between study arms. In addition, data on potential confounding factors including number of people in the house, burning incense [20] will be collected and corrected for use in the analysis. The interventions will be closely monitored for quality and durability and to document any bias among the villages.

### Interventions

**Fig. 2:**
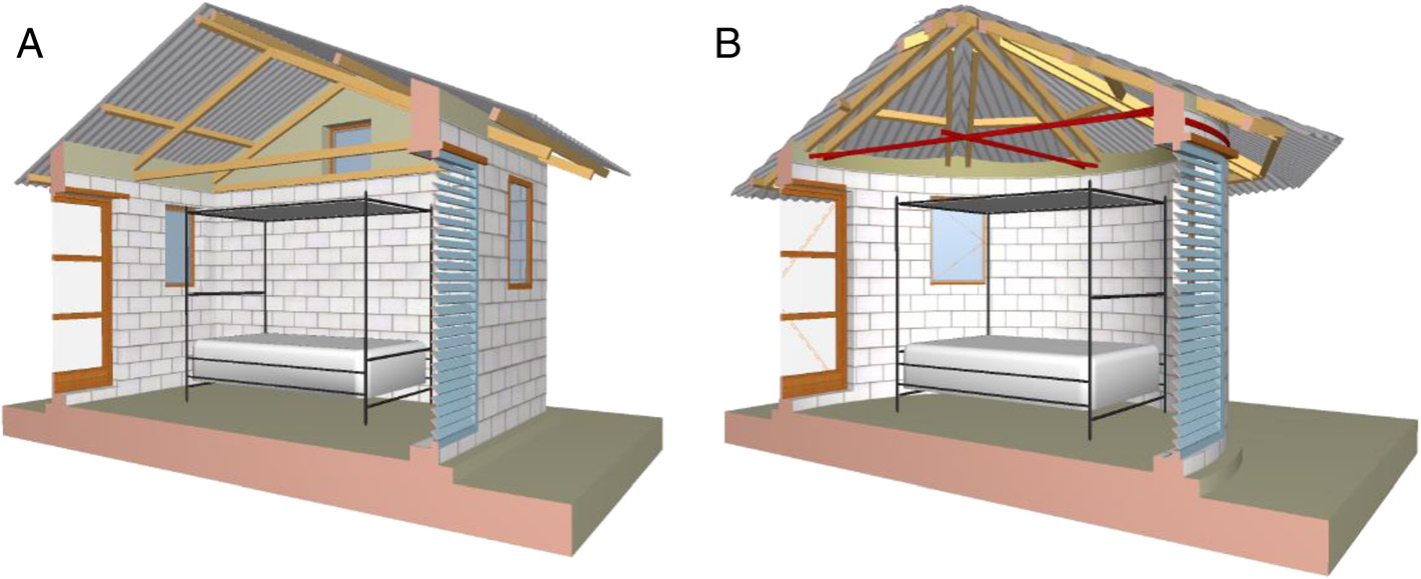
Prototype rectangular-plan (**a**) and circular-plan thatched-roof houses (**b**)

Enrolled houses will all have thatched roofs and mud walls in good condition, and the occupants, provided with a sufficient number of LLINs (Olyset, Sumitomo Chemical) to cover all sleeping places. At the time of donation, we will follow national guidelines to encourage their correct use, as this is the current best practice. In the intervention arm, represented by modern housing, we propose to modify existing rectangular-plan and circular-plan thatched roof houses, so they will have metal roofs, closed eaves [19], and screening on the doors and windows (Fig. 2, *n* = 400).The control arm, representing traditional houses, will be left with thatched roofs and open eaves (*n* = 400) until the end of the study. A metal-louvered screened door will be installed in the front of the house, and a wooden-screened door, at the back [21]. Households will be enrolled, and the interventions put in place from February 2015 to June 2016. Homeowners will be encouraged to keep their doors closed at night.

### Clinical data collection and patient treatments

The baseline clinical survey of all study children will take place in May/June 2016 and will determine malaria infection, splenomegaly, and anemia. Clinical follow-up will start in June 2016, and cover both rainy seasons (June to December 2016 and 2017). Incidence of clinical malaria will be determined by ACD during twice weekly house visits from June to November each year [20]. Clinical respiratory disease will be determined at the same time. Clinical surveys of all study children will be repeated at the end of each rainy season (November/December) and during June 2017. Surveys to measure damage to the interventions and the integrity of the control houses will be carried out annually. Surveys to measure LLIN use by all study house residents will be made in July and October each year. The final clinical survey of the child cohort will take place in December 2017.

No treatment will be administered by the study personnel. However, any patient diagnosed with malaria or suspected to have pneumonia or other illnesses will be referred to the local health system for treatment according to the Gambian National Guidelines.

### Clinical evaluations

The main morbidity outcome will be the incidence of clinical disease assessed by ACD; data from this will provide the primary endpoint of the study. The axillary temperature will be taken twice weekly and if ≥ 37.5 °C, a rapid diagnostic test (Paracheck Pf, Orchid Biomedical Systems, Goa, India) for malaria will be made, and the breathing rate will be checked. The presence of a cough and either a raised age-specific respiratory rate or chest indrawing will also be assessed in children deemed to be unwell by the parent or guardian. Serious adverse events (SAE) will be recorded during both transmission seasons. Sick children will receive referral notes to the nearest health post/facility, and treatment will be documented at the next biweekly visit.

During the cross-sectional surveys, clinical surveys will be done of all study children, who will be examined for general health and the presence of an enlarged spleen. A finger prick sample will be taken to measure the blood hemoglobin immediately using a spectrophotometer (HemoCue, Ängelholm, Sweden) and to prepare thick smears for later detection and quantification of *Plasmodium* parasites. Blood films will be stained in the field by project staff, transported to the laboratory, and read independently by two microscopists blinded to the identity of the child. Any discrepancy will be resolved by a third senior microscopist. Children with sickness or disease will be referred for treatment.

### Entomological evaluations

Indoor mosquito collections made using Centers for Disease Control (CDC) light traps will be used to estimate the potential exposure to malaria vectors and will be carried out once a month for 6 months from June to December in 2016 and 2017 in the bedrooms of the study children. Mosquitoes will be identified by microscopy, and the numbers of *An. gambiae* s.l. and other species, recorded. The presence of sporozoites in *An. gambiae* s.l. will be identified using an enzyme-linked immunosorbent assay [22], and *An. gambiae* s.l. females, typed to species by PCR [23].

### Economic and social science evaluations

Data on incremental costs of house improvements and their sources will be collected alongside the interventions. Incremental benefits due to malaria cases averted will be estimated based on malaria treatment cost data collected from selected health facilities. The acceptability of the intervention will be captured during the trial using (1) observations and informal conversations during the house modification process, (2) photo-voice (a participatory action research technique enabling people to record and reflect on their concerns, promote critical dialogue, and reach policy makers) [24], and (3) focus group discussions. These activities and their outputs will contribute to the process of stakeholder engagement and will be used to develop an end-of-trial acceptability questionnaire survey. To design potential strategies for scale-up, we will identify and engage with key local, national, and international level stakeholders, involving them in discussions and decisions on the appropriate innovation design and consideration of the implications of the results and creating a driving team to identify mechanisms for expansion and institutionalization while acting as advocates for the innovation.

### Safety considerations

No potential pharmacological intervention or treatment by project staff is proposed in this study. However, the possibility remains that our modified housing may increase the risk of acute respiratory infections. We will therefore assess the study children for respiratory disease at each study visit, i.e., twice weekly during the transmission season. Since respiratory infections are common in children in this area [25], we selected this age group as the most sensitive indicator group. Adverse events, whether attributed or not to improved housing, will be collected. If a participant develops a SAE during the course of the study, this will be reported by the field staff to the study clinician within 2 days of the start of the SAE. A SAE is defined as any adverse event that results in death, is life threatening, requires hospitalization or prolongation of existing hospitalization, or results in disability/incapacity. The study clinician will record and manage the SAE in accordance with the MRC Unit standard procedures (SOP-CTS-009 for “Recording, Management and Reporting of Adverse Events”). The study clinician will report the SAEs to the Chief Investigator, who will report these regularly to the Data Safety and Monitoring Committee and Study Steering Committee (SSC; see supplementary material for roles and responsibilities). All SAEs will be followed up until resolution.

### Handling of drop-outs/withdrawals

Participants are free to withdraw from the study at any time without giving a reason. In the unlikely event that homeowners require their house to be returned to the pre-intervention state, we will remove the items we have added and replace the metal roof with a thatched one. Withdrawals may also occur if a study subject has any clinically significant AE, laboratory abnormality, intercurrent illness, or other medical condition or situation that occurs such that continued participation in the study would not be in the best interest of the participant. No replacements will be made during the surveillance period in either year, but in May 2016, children who have withdrawn or are no longer in the study for any reason will be replaced by a child of a similar age from the same house. If a household withdraws consent, no further follow up will be made in that household. If the house was participating in the entomology collections, it will be replaced by a neighboring house of the same type, if possible.

### Study endpoints

#### Clinical

The primary clinical endpoint will be the incidence of clinical malaria, which is determined by active case detection (ACD) and defined as a body (axillary) temperature of ≥ 37.5 °C, together with the presence of *P. falciparum* parasites detected by microscopy.

The secondary clinical endpoints will be (1) *P. falciparum* parasite rates, (2) prevalence of splenomegaly, (3) prevalence of anemia, and (4) an incidence of respiratory infection, measured as a cough and either a raised age-specific respiratory rate or chest indrawing.

#### Entomological

The primary entomological endpoint will be the mean number of female *An. gambiae* s.l./light trap/night.

The secondary entomological endpoint will be the estimated entomological inoculation rate (EIR) in each study arm (i.e., the mean number of sporozoite infective bites/child/season).

#### Economic and social sciences

The primary economic and social sciences endpoints will be the mean and median costs of house improvement interventions/house improved and the mean and median costs of treating one case of childhood malaria.

The secondary economic and social sciences endpoints will be the percentage of home owners who agreed to house modifications in the intervention arm, key observations and discussions on house modifications with villagers and builders in the trial, trends in the types and quantities of housing materials imported/exported, the construction skills developed during at least the previous 5 years in the country, and a road map for scaling up of the housing modifications.

### Sample size rationale

#### Clinical

**Fig. 3:**
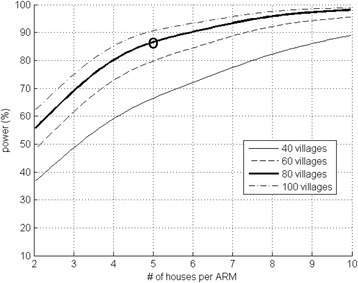
Sample size calculations

The combination of closed eaves and screening on the door reduces house entry of malaria mosquitoes by 65 % [26], and we anticipate a similar proportional reduction in malaria incidence in the proposed study but consider that a reduction of at least 35 % would not only be of public health importance but also good value for the money. We simulated incidence data allowing for within-house correlation between the years, based on incidence data collected using passive case detection from the proposed study area in 2010 (0.0468 and 0.0442 malaria episodes per child month at risk, CMAR) and 2011 (0.0321 and 0.0341 CMAR). The number of cases were inflated twofold, as we will use active case detection (ACD). Correlated event data were generated from a bivariate Poisson distribution to give random simulated correlated counts for each house over the two years. Each village was assumed to contain an equal number of houses in the control and intervention arm. Fifty sets of 1,000 data points were simulated for each of the two to ten houses in the 40 to 100 villages. The significance of the difference in incidence rate between the control and the intervention arms was quantified using a generalized estimating equation (allowing for the within-house correlation) with a log link for each of the data sets and house and village parameters. The proportion of each set of 1,000 simulations with a significant *p* value of less than 5 % for the difference between the control and the intervention arm was the estimated power. This shows that when following one child per house for 2 years, five houses in 80 villages are required in each study arm (black circle, i.e., 400/arm) to detect a 35 % reduction in malaria cases with more than 80 % power at the 5 % significance level (Fig. 3).

#### Entomological

We postulate that metal-roofed houses, closed eaves, and screening on the doors will reduce indoor collections of malaria vectors by at least 50 %. Based on a study using light traps in the proposed study area in 2011, we expect that the mean number of female *An. gambiae* s.l. per trap will be 2.11 (SD = 1.80). In order to demonstrate a 50 % reduction in indoor-entering mosquitoes (i.e., unfed *An. gambiae*) associated with housing interventions, with 80 % power at the 5 % level of significance, we would require 47 houses in each arm of the study respectively. Since the number of mosquitoes rise and fall during the rainy season, we propose to sample from each house six times each year. To allow for loss to follow-up during the study period, we propose to conduct entomological monitoring in 60 houses in each arm of the study each year.

### Data handling and record keeping

The demographic data will be recorded by fieldworkers, and the clinical data by study nurses on standardized data forms. Each child in the cohort will be identified by a unique identification number. All forms and datasets will identify participants by these numbers, and names will not be entered. The study will follow the MRC Unit The Gambia’s specific policy to maintain anonymity of study participants. Personal identifiers will be removed from the transcripts of interviews, and discussions with participants will be identified only through a study identification number. Transcribe and translated transcripts will be entered into NVivo for management and analysis. Participants in the photo-voice exercise will be specifically requested and trained not to take photographs containing images of identifiable people, and any photographs containing such images will not be entered into the NVivo database. Thus all data will be anonymized at data entry, including those participating in the subsidiary studies (economic and perceptions of the housing intervention). Databases will be password protected and accessible only to authorized personnel. All documents will be securely stored in locked filing cabinets and accessible only to authorized personnel.

### Source documents and access to source data

The PI will maintain appropriate medical and research records for this study in compliance with the principles of good clinical practice and regulatory and institutional requirements for the protection of confidentiality of participants. The study team members will have access to records. The authorized representatives of the sponsor, the ethics committee(s), or regulatory bodies may inspect all documents and records required to be maintained by the investigator, including but not limited to medical records (office, clinic, or hospital) for the participants in this study. The clinical study site will permit access to such records. The results of the study will be made publicly available. Blood slides and mosquito DNA will be stored and made available for future studies.

### Analytical plan

A per-protocol and an intention-to-treat analysis will be conducted.

***Outcome 1—malaria morbidity*.** Protective efficacy against clinical malaria will be determined by comparing incidence rates of clinical malaria between arms. After any treatment for malaria, the child will not be considered at risk for 4 weeks, and this period will be censored. History of travel away from the household will be captured by twice-weekly surveys during the malaria season, and the time at risk will be censored for such periods. An initial unadjusted analysis will be based on comparisons of the incidence rates between the two arms. Formal analysis will use a mixed effects Poisson (or negative binomial) model to test the difference in incidence rate between the two arms, allowing for the repeated measurements within house and within village and the effect of year. With multiple control and intervention houses within each village, the intervention * village interaction can also be quantified. Possible confounders such as age of child, gender, ethnicity, and rainfall will be tested using the mixed effects model.

A comparison of the time to the first episode of clinical malaria and repeat episodes of clinical malaria will be examined using a survival analysis approach. Initial analyses will be based on Kaplan-Meier curves with further adjustment for confounders performed using a Cox regression model.

***Outcome 2—malaria transmission.*** Differences in malaria transmission experienced in the two groups will be made by comparing the mean number of mosquitoes (as a proxy for transmission) caught indoors in houses between the intervention groups. Generalized estimating equations will be used to estimate differences in the numbers of indoor-resting mosquitoes, adjusting for repeated measures within houses and possible covariates.

***Outcome 3—incremental cost-effectiveness ratio.*** The incremental cost-effectiveness ratio of averting a clinical case of malaria by home improvement interventions will be calculated through probabilistic decision tree analysis. The health impact will be also expressed in terms of disability-adjusted life years.

***Outcome 4—housing market supply.*** Data on the types and quantities of construction material produced and imported during at least the previous 5 years, as well as of construction skills developed within the country, will be analyzed against the resources used within the trial. Time-trend analysis will be also performed to generate future predictions of the quantities potentially available in the next few years, while controlling for confounding factors.

### Blinding

Given the nature of the intervention, it is impossible to conduct this study in a fully blinded manner, but those parts of the data collection that can be blinded will be. Observer bias will be reduced where feasible. Blood films will be read by microscopists blinded to the identity and intervention status of the subjects. Mosquito collection bias will be reduced by using standard light traps that do not rely on the ability of the fieldworker to collect specimens. Trap catches will not be examined and analyzed by those who collected them but by different technicians who will not know the trap location. Datasets will only be unblinded after all those critical for the listed primary and secondary endpoints have been locked.

### Trial oversight

An SSC will provide overall supervision of the trial and ensure that the trial is conducted to the standards set out in the Medical Research Council’s Guidelines for Good Clinical Practice (https://www.mrc.ac.uk/documents/pdf/good-clinical-practice-in-clinical-trials/). In particular, the SSC will concentrate on the progress of the trial, adherence to the protocol, patient safety, and the consideration of new information, and the SSC will formally report to the sponsor (Durham University). The Data Monitoring Committee will determine if additional interim analyses of trial data should be undertaken, and assess any additional safety issues that may arise during the study. They will ensure the safety, rights, and well-being of the study participants and will report to the SCC at regular intervals. The roles and responsibilities of the steering committees are described in Additional file 4. The completed SPIRIT checklist is described in Additional file 5.

### Ethical approval

This study is being conducted in accordance with the principles set forth in the ICH Harmonised Tripartite Guideline for Good Clinical Practice and the Declaration of Helsinki in its current version, whichever affords the greater protection to the participants. It was approved by The Gambia Government/MRC Joint Ethics Committee on 29 October 2014 (ref: SCC 1390v3) and the School of Biological and Biomedical Sciences Ethics Committee, Durham University, on 1 December 2014 (ref: SBBS/EC/1401/RooPfs 12 09 14). Approval for any important protocol modifications will be sought from both ethics committees.

## Discussion

Most malaria-control interventions are based on the use of chemicals. While drugs and vaccines are used to protect people from the disease, insecticides are used to protect people from potentially infective mosquito bites. The Roo*Pf*s study is novel because we are not using chemicals as an intervention. This is important because the major threat to global malaria control today is from strains of vectors that are resistant to the insecticides used for treating bed nets or spraying indoors [3]. Since vector control with LLINs and IRS are the mainstay of malaria control today, supplementary tools are needed to combat the threat from insecticide-resistant mosquitoes. Improved housing is one potential tool that could be used for malaria control and is one that will be effective against insecticide-resistant vectors. This intervention could be used to limit malaria in areas where malaria has been eliminated and where LLINs and IRS are no longer used.

The quality of housing is improving across many parts of sub-Saharan Africa. Despite the global economic recession, Africa’s economy is the fastest growing of any continent (Economist 2013, Africa rising – a hopeful continent, 2 March), and the economies of sub-Saharan Africa are anticipated to grow at a rate of 6 % per annum over the next decade. Such rapid development is leading to marked changes in the housing stock with the traditional thatched houses disappearing in many areas. With a rapidly growing population, the need for further housing is pressing, and this provides an opportunity for new housing construction that will protect the occupants from vector-borne diseases such as malaria.

A recent systematic review suggests that improved housing could reduce the amount of malaria infection and disease by half [12]. Our present study is the first to test the assumption that modern housing will reduce the incidence of clinical episodes of malaria in a randomized controlled study. In the proposed study area, the incidence of malaria as measured by passive case detection is relatively low, with 0.2–0.3 children experiencing a malaria episode during the transmission season [27]. Since clinical episodes of malaria are our primary outcome measure, we will use ACD to maximize the number of cases. Study children will be visited twice weekly by project staff, which should increase the number of children diagnosed with clinical malaria. In a pilot study in The Gambia, five cases of malaria were identified by weekly surveillance, and eight, by daily surveillance, although this difference was not statistically significant [28]. We have selected children aged 6 months to 13 years for two reasons. First, the lower limit was selected because few children experience a clinical episode of malaria in the first 6 months of their life [29–32]. Secondly, since the level of malaria transmission of malaria is relatively low at around 0.29–2.44 infective bites per year for individuals sleeping without a LLIN, the burden of clinical malaria has moved from those under 5 years old to older age groups.

The potential risk exists to study children that screening houses from mosquitoes will reduce the air circulation in the house, making it hotter, and perhaps increasing the risk of respiratory disease. We think this is unlikely but will record respiratory disease in the study cohort. It should be appreciated that all study children will be have access to LLIN at the start of the study, and that the nets limit ventilation sharply [33]. Indeed, it may be that the screening is so effective at reducing mosquito house entry that some people living in screened homes may stop using their nets [13]. In these instances, air movement across subjects sleeping without a net will be greater than those sleeping under a net.

Study subjects will benefit from this study by having a desired commodity: a metal-roofed and screened house. Children will also have a health check at the surveys. However, a number of limitations exist for the use of improved housing to control malaria. First, we do not know whether the housing interventions will be acceptable to local people. Second, we do not know how durable the interventions will be. Third, this intervention is most likely to be introduced outside the health sector, so innovative new pathways to scale up will need to be developed. Finally, we are unaware of the costs of this intervention but recognize that they are likely to be greater than conventional vector-control strategies. All of these issues will be addressed in ancillary studies being carried out in parallel with the randomized controlled study. Furthermore, improved housing is not an “evolution proof” intervention. If vectors adapted to entering houses are prevented from doing so, it is possible that vectors will be selected for biting outdoors, possibly early in the evening, when they are most likely to feed. Such changes in behavior have been seen when LLINs or IRS have been used on a large scale [34]. This emphasizes the importance of using several interventions at once, against the vector and the parasite, so that transmission can be minimized and malaria eliminated in the window before such adaptive changes occur. This protocol describes a randomized controlled study to measure the impact of improved housing against malaria in Gambian children.

### Protocol version

17 July 2015, version 2.0.

### Status

The trial is currently recruiting.

## Abbreviations

ACD, active case detection; AE, adverse event; CDC, Centers for Disease Control and Prevention; CRF, case report form; CV, coefficient of variation; EIR, entomological inoculation rate; GCP, good clinical practice; Hb, hemoglobin; ICH, International Conference on Harmonisation of Technical Requirements for Registration of Pharmaceuticals for Human Use; ID, identification number; LLIN, long-lasting impregnated nets (permethrin-treated); PCR, polymerase chain reaction; *Pf*PR, *Plasmodium falciparum* parasite rates; PI, principal investigator; RDT, rapid diagnostic test; SSA, sub-Saharan Africa; SAE, serious adverse event; WHO, World Health Organization; WHOPES, WHO Pesticide Evaluation Scheme

## Additional files

Additional file 1: Household information sheet and consent form. (DOCX 97 kb) Additional file 2: Child information sheet and consent form. (DOCX 25 kb) Additional file 3: Schedule of enrolment, interventions and assessment. (DOC 49 kb) Additional file 4: Roles and responsibilities of overseeing committees. (DOCX 29 kb) Additional file 5: SPIRIT checklist: recommended items to address in a clinical trial. (DOC 119 kb)
