# Supplementary material for: The RooPfs study to assess whether improved housing provides additional protection against clinical malaria over current best practice in The Gambia: study protocol for a randomized controlled study and ancillary studies
Source: Trials. 2016 Jun 3;17:275. doi: 10.1186/s13063-016-1400-7 (PMC4891825; doi:10.1186/s13063-016-1400-7)
Supplement: Additional file 1: — Household information sheet and consent form. (DOCX 97 kb) [file 13063_2016_1400_MOESM1_ESM.docx]

**HOUSEHOLD INFORMATION SHEET**

| Version | 3.0 | Date | 23^rd^ March 2015 |
| --- | --- | --- | --- |

Study Title:

| SCC and Protocol: | **Can improved housing provide additional protection against clinical malaria over current best practice? A household-randomised controlled study. Code name Roo*Pf*s.** |
| --- | --- |

**Sponsor**: Durham University

What is informed consent?

You are invited to take part in a research study called ‘Roofs’. Participating in a research study is not the same as getting regular medical care. The purpose of regular medical care is to improve one’s health. The purpose of a research study is to gather information. It is your choice to take part and you can stop any time.

Before you decide you need to understand all information about this study and what it will involve. Please take time to read the following information or get the information explained to you in your language. Listen carefully and feel free to ask if there is anything that you do not understand. Ask for it to be explained until you are satisfied. You may also wish to consult your spouse, family members or others before deciding to take part in the study.

If you decide to join the study, you will need to sign or put a thumbprint on a consent form saying you agree to be in the study. You will receive a copy of this.

Why is this study being done?

Malaria causes a lot of illness in children in The Gambia and we want to find better ways of protecting people against this disease. People get malaria when bitten by one type of mosquito. The mosquito spits the malaria germ into you making you sick.

What is the new intervention?

In this study we want to find out if, in addition to everyone sleeping under insecticide-treated bednets, improving houses is an effective way of protecting people against malaria.

The improvements we want to do are:

[*show photograph or drawing of new house design*]

1. Replace a thatched roof with a metal one
2. Close the gap between the wall and roof with mortar
3. Put screened windows in the gables to make the house cooler
4. Screen the doors and window to keep out mosquitoes

We need to compare houses with the new intervention and ones without.

*[Some houses in this village have been selected to receive the new intervention in the 20152016 dry season and some to receive it after December 2017. Check the selection list and either read A or B].*

**What will happen if you agree to join**:

***A. House selected to receive the new intervention in the 2015 / 2016***

- - - 1. Your house has been selected for building improvements.
      2. If you agree, a team of builders will make changes to your house in the first half of 2015 / 2016.
      3. When building we expect you to make sure that:
      - All the people leave the houses before the work starts
      - All the animals are tied away from the house
      - All the household items, for example furniture, water, food, cooking utensils, toys, wall hangings, are removed from the house
      - All furniture and water-jars should be removed from the house before building. If this is not possible, they should be moved to the middle of the room and covered.
      1. The building work will be carried out by builders trained by the study under the supervision of the project. All materials from outside the village and builders costs will be paid for by the project.
      2. We may also put two small measuring devices on your bedroom wall to record how hot and damp the room gets.
      3. We will seek permission to enroll one child from your house to join the malaria cohort
      4. We will seek permission to inspect and record the house improvements occasionally.
      5. We may seek your permission to ask you questions about the house improvements.
      6. We may seek permission to position a light trap in one of your rooms once a month during the rainy season.

***B. House selected to receive the new intervention after December 2017***

1. If you agree, you will be offered free home improvements for your house after the end of the rainy season in 2017.
2. If you agree to join the study now you will receive insecticide-treated bednets and we ask you to ensure that everyone in your household has access to one.
3. We may also put two small measuring devices on your bedroom wall to record how hot and damp the room gets.
4. We will seek to permission to enroll one child from your house to join the malaria cohort.
5. We may seek permission to position a light trap in one of your rooms once a month during the rainy season.
6. We may also seek permission to measure the airflow and dust in your house using some machines.

What harm or discomfort can you expect in the study?

You will have to leave your house during the days we are building. The building work will take about 3 days and you can go back into your house each evening but the roof may not have been completed before day 2 or 3. The disturbance to your daily activities will be kept to a minimum.

What benefits can you expect in the study?

We hope that the changes made to your house will be useful to you and your family. The results of the study will help us learn how best malaria can be controlled.

Will you be compensated for your participation in the study?

You will not get paid for participation in the study.

What happens if you refuse to participate in the study or change your mind later?

You are free to participate or not in the study and you have the right to stop participating at anytime without giving a reason. In case you decide to withdraw your participation during the study we not collect any more information from your house, but any information already generated will be kept.

What compensation will be available if you are injured during the study?

We will be responsible for providing treatment caused by procedures of the research study.

How will your information be kept and who will be allowed to see it?

All information that is collected in the course of the study will be kept strictly confidential. Your personal information will only be available to the study team members and might be seen by some rightful persons from the Ethics Committee, Government authorities and sponsor.

Who should you contact if you have questions?

If you have any queries or concerns you can contact Dr Margaret Pinder on [to be added] and you can always call the personal numbers of the study staff given to you.

Please feel free to ask any question you might have about the research study.

## Who has reviewed this study?

This study has been reviewed and approved by a panel of scientists at the Medical Research Council and the Gambia Government/MRC Joint Ethics Committee, which consists of scientists and lay persons to protect your rights and wellbeing.

**Consent Form**

Name of participant__________________________________________

(Printed name of participant)

Household number|__|__|__| Compound Code|__|__|__|Village Code|__|__|__|

Village Name ____________________Compound Head_______________________

I have read the written information **OR**

I have had the information explained to me by study personnel in a language that I understand,

and I

- confirm that my choice to participate is entirely voluntarily,
- confirm that I have had the opportunity to ask questions about this study and I am satisfied with the answers and explanations that have been provided,
- understand that I grant access to data about my house to authorised persons described in the information sheet,
- have received time to consider to take part in this study,
- agree to take part in this study.

|  |  |  |
| --- | --- | --- |

| Participant’s signature/ thumbprint* |  |  |  |  |
| --- | --- | --- | --- | --- |
|  |  |  | Date (dd/mmm/yyyy) Time (24hr) | |
|  |  |  |  | |
| Printed name of witness* |  | | | |
| Printed name of person obtaining consent |  | | | |
| **I attest that I have explained the study information accurately in the household information sheet, and this was understood to the best of my knowledge by the participant. He/she has freely given consent to participate ***in the presence of the above named witness (where applicable).** | | | | |
| Signature of person obtaining consent |  |  |  | |
|  |  |  | Date (dd/mmm/yyyy) Time (24hr) | |
| ** Only required if the participant is unable to read or write.* | | | | |
