# Supplementary material for: The RooPfs study to assess whether improved housing provides additional protection against clinical malaria over current best practice in The Gambia: study protocol for a randomized controlled study and ancillary studies
Source: Trials. 2016 Jun 3;17:275. doi: 10.1186/s13063-016-1400-7 (PMC4891825; doi:10.1186/s13063-016-1400-7)
Supplement: Additional file 2: — Child information sheet and consent form. (DOCX 25 kb) [file 13063_2016_1400_MOESM2_ESM.docx]

**Can improved housing provide additional protection against clinical malaria over current best practice? A household-randomised controlled trial.**

**Code name RooPfs.**

**SCC/EC 3019/2014**

**Child Information Sheet**

**Responsible scientists:**

Dr Margaret Pinder, MRC Basse, Professor Umberto D’Alessandro, MRC Fajara, and Professor Steve Lindsay, Durham University, U.K.

**Introduction:**

Your child is invited to take part in a research study. Before you decide it is important for you to understand why the research is being done. Allow me to explain what the study is all about and what it will involve. Please take time to listen to the information carefully. Ask us if there is anything that is not clear or understood.

**Purpose:**

Malaria causes a lot of illness in children in The Gambia and we want to find better ways of protecting people against this disease. People get malaria when bitten by one type of mosquito. The mosquito spits the malaria germ into you making you sick.

In this study we want to find out if improving your home in a way that will reduce the number of mosquitoes coming inside is an effective way of protecting people against malaria. You have already agreed to have modifications made to your house either in the next few months or in two years’ time. We would now like to ask your permission for your child to take part in the study

**What will happen if you agree for your child(ren) to participate**:

1. We will take a picture of your child and give them an identity badge with their study number. We will ask you to keep this safely.
2. We will check the health of your child at the survey today and in June 2015, December 2015 and December 2016. We request permission to take a finger-prick sample (2-3 small drops) of blood from your child. The blood taken will be used to check if the blood is weak (anaemia) and some will be sent to the laboratory so we can look for malaria parasites. In addition, the specially trained nurse-field assistant will feel your child's tummy to measure the size of their spleen (a special gland that normally hides underneath the ribs). If your child is sick at these surveys we will examine the child and if they need treatment we will give you a referral slip so you can obtain the correct treatment without cost to you at the nearest health post or facility.
3. Also, during the rainy season in 2015 and 2016, your child will be visited by a MRC nurse field assistant twice each week to check their health. The field assistant will take the child’s temperature and if the child has a hot body the field assistant will take a single finger-prick blood sample (2-3 small drops of blood) from your child to test whether they have malaria or not. If the child has malaria or another health problem including a chest problem that needs treatment they will be given a referral slip so you can obtain the correct treatment at nearby health post or facility. This will not cost you any money.
4. If your child is not present at the visit the field assistant will ask you, or your family, if the child has travelled and record the days that the child is absent. This is only so that we know have much of the rainy season the child was living in this house.
5. At each visit the field assistant will record if your child is using an insecticide treated bed net.

**Risks:**

The taking of blood may cause a little discomfort from the stick of the lancet.

**Benefits:**

If your child becomes sick with malaria or chest problems during the rainy season or have anaemia at the time surveys, they will receive free treatment.

The results of the study will help us learn how best malaria can be controlled.

**Participation:**

You have the right not to join the study and you can leave the study at any time without penalty or loss of benefits to which you and your child are otherwise entitled.

**Confidentiality:**

Your child or children’s individual information will be kept private.

**Payment:**

There is no payment for joining in this study.

**Research sponsor:**

This research is sponsored by the MRC UK, the Wellcome Trust and the UK Department for International Development.

**Institutional Approval**:

This study has been approved by the Joint MRC and Gambian Government’s Ethics Committee.

**Please feel free to ask questions**

**Can improved housing provide additional protection against clinical malaria over current best practice? A household-randomised controlled trial.**

**Code name RooPfs.**

**SCC/EC 3019/2014**

**Child Consent Form**

I, ………………………………………………………………(name of parent or guardian) consent to

my child ………………………………….…………..(name of child) to take part in the **RooPfs** research study.

I, ………………………………………………………………(name of study subject, if over 6 years of age) agree to participate in the research project.

The purpose of the research study has been explained to me and I have been given the opportunity to ask questions concerning this study. Any such questions have been answered to my full satisfaction. Should any further questions arise concerning this study I may contact Dr Margaret Pinder or the Study Physician (to be appointed), MRC Basse.

I also understand that I may withdraw from this study at any time. I may do this orally or in writing and I will not face any penalty if I do so.

Signature or thumbprint of the parent or guardian of the subject……………………………………………

Date…………………………………

Signature or thumbprint of study subject (if over 6 years of age)…………………………………………..

Date…………………………………

ID No……………………… Village Name …………………………………………………………..Village Code………… Compound Code………………………….. Compound Head………………………………………………………………..

Witness name……………………………………………….Signature………………………………………………….

Date……………………………………

Person obtaining consent: Role…………………………………Name……………………………………Signature…………………………………

Date……………………………………
