# Supplementary material for: The RooPfs study to assess whether improved housing provides additional protection against clinical malaria over current best practice in The Gambia: study protocol for a randomized controlled study and ancillary studies
Source: Trials. 2016 Jun 3;17:275. doi: 10.1186/s13063-016-1400-7 (PMC4891825; doi:10.1186/s13063-016-1400-7)
Supplement: Additional file 3: — Schedule of enrolment, interventions and assessment. (DOC 49 kb) [file 13063_2016_1400_MOESM3_ESM.doc]

Figure 1. Schedule of enrolment, interventions, and assessments for Roo*Pf*s randomized clinical study.*

|  | **STUDY PERIOD** | | | | | | |
| --- | --- | --- | --- | --- | --- | --- | --- |
|  | **Enrolment** | **Allocation** | **Post-allocation** | | | | **Close-out** |
| **TIMEPOINT**** | ***Dec 2014-May 2016*** | **Jan 2015-May 2016** | ***Jun 2016*** | ***Dec***  ***2016*** | ***Jun***  ***2017*** | ***Dec 2017*** | ***Dec 2017*** |
| **ENROLMENT:** |  |  |  |  |  |  |  |
| **Eligibility screen** | X |  |  |  |  |  |  |
| **Informed consent** | X |  |  |  |  |  |  |
| ***[List other procedures]*** | X |  |  |  |  |  |  |
| **Allocation** |  | X |  |  |  |  |  |
| **INTERVENTIONS:** |  |  |  |  |  |  |  |
| ***Improved housing & long-lasting insecticidal nets*** |  |  |  |  |  |  |  |
| ***Traditional housing & long-lasting insecticidal nets*** |  |  |  |  |  |  |  |
| **ASSESSMENTS:** |  |  |  |  |  |  |  |
| ***Presence of malaria parasites, presence of an enlarged spleen, haemoglobin*** |  |  | X | X | X | X |  |
| ***Active case detection of clinical malaria*** |  |  |  |  |  | . |  |
| ***Entomological surveillance*** |  |  |  |  |  |  |  |

*Recommended content can be displayed using various schematic formats. See SPIRIT 2013 Explanation and Elaboration for examples from protocols.

**List specific timepoints in this row.
