# Supplementary material for: The RooPfs study to assess whether improved housing provides additional protection against clinical malaria over current best practice in The Gambia: study protocol for a randomized controlled study and ancillary studies
Source: Trials. 2016 Jun 3;17:275. doi: 10.1186/s13063-016-1400-7 (PMC4891825; doi:10.1186/s13063-016-1400-7)
Supplement: Additional file 4: — Roles and responsibilities of overseeing committees. (DOCX 29 kb) [file 13063_2016_1400_MOESM4_ESM.docx]

# Overseeing Committees for Roo*Pf*s

## Study Steering Committee (SSC)

These conditions are based on the MRC Guidelines for Good Clinical Practice in Clinical Trials (Update 2009).

### Role of the SSC

The role of the SSC is to provide overall supervision of the trial and ensure that the trial is conducted to the rigorous standards set out in these MRC Guidelines for Good Clinical Practice. In particular, the SSC should concentrate on progress of the trial, adherence to the protocol, patient safety and the consideration of new information, and will formally report to the Sponsor (Durham University). Applicants should submit a proposal for the membership of the SSC with their full application to the MRC who will agree the final membership. The membership should be limited and include an independent Chair (not involved directly in the trial other than as a member of the SSC), not less than two other independent members plus the Chief Investigator and one or two Principal Investigators, with additional experts if required. The Trial manager/coordinator, statisticians etc. should attend meetings as appropriate. An observer from the MRC and Host Institution or sponsoring organisation if different, should be invited to attend all Steering Committee meetings.

### Meetings

A meeting of the SSC should be organised by the CI before the start of the trial to approve the final protocol, which should then be sent for review by a Research Ethics Committee, and any other applicable regulatory body. After that the SSC should meet at least annually although there may be periods when more frequent meetings are necessary and may be called either by the Chair of the

SSC or the CI. Responsibility for calling for and organising Steering Committee meetings lies with the CI, following consultation with other Principal Investigators. However, there may be occasions when the MRC will wish to organise and administer these meetings for particular trials. Papers for meetings should be circulated well in advance of the meeting rather than tabled and an accurate minute of the meeting should be prepared by the CI and agreed by all members. The minutes must be available to MRC on request.

### Terms of reference

1. to monitor and supervise the progress of the Roo*Pf*s study towards its interim and overall objectives;
2. to review at regular intervals relevant information from other sources (eg, other related trials);
3. to consider the recommendations of the Data Monitoring Committee;
4. to report to the sponsor on progress of the trial, and if necessary to MRC as funder.
5. to advise CI, Sponsor and MRC as funder on publicity and the presentation of all aspects of the trial. Focus on the progress of the trial, adherence to the protocol, patient safety and the consideration of new information.

### Membership

Applicants should submit a suggested membership with their full application. The relevant Board(s) will decide on the final membership of the SSC. The membership should be limited and include an **independent** Chair (not involved directly in the trial other than as a member of the SSC), not less than two other independent members plus the Chief Investigator and one or two Principal Investigators and additional experts if required. Where possible, membership should include lay/consumer representation. The Trial manager/co-ordinator, statisticians etc. should attend meetings as appropriate. An observer from the MRC and Host Institution or sponsoring organisation if different, should be invited to attend all Steering Committee meetings.

### Independent members

Prof Christian Lengeler (Chair) Christian.Lengeler@unibas.ch

Dr Mike Macdonald (USAID) macdonald@macito.net

Dr Mari Stephens (Habitat for Humanity) MaStephens@habitat.org

### Funder representative

Dr Meriel Flint (MRC) Meriel.Flint@headoffice.mrc.ac.uk

### Host organisation representative

Prof Umberto D’Alessandro (Chair, Director, MRC Unit, The Gambia) udalessandro@mrc.gm

### Chair of DMC (invited to first meeting)

Prof Brian Faragher (Statistician, Liverpool Sch. of Tropical Medicine) Brian.Faragher@lstmed.ac.uk

### Study members

Prof Steve Lindsay (Chief Investigator) S.W.Lindsay@durham.ac.uk

Dr Margaret Pinder (Principal investigator) mpinder@mrc.gm

Mrs Rachel Simpson (Overseas Projects Administrator) Rachel.Simpson@durham.ac.uk

### Guidance notes

#### Meetings

A meeting of the SSC should be organised by the CI before the start of the trial to approve the final protocol, which should then be sent for review by a Research Ethics Committee (REC), and any other applicable regulatory body. After that the SSC should meet at least annually although there may be periods when more frequent meetings are necessary and may be called either by the Chair of the

SSC or the CI. Responsibility for calling for and organising Steering Committee meetings lies with the CI, following consultation with any other Principal Investigators. Papers for meetings should be circulated well in advance of the meeting rather than tabled and an accurate minute of the meeting should be prepared by the CI and agreed by all members. The minutes must be available to

MRC on request.

#### Data Monitoring Committee

The SSC should establish a data monitoring committee and invite the chair of the DMC to its first meeting. The DMC should meet regularly to view the data and the results of any interim analyses, particularly in blinded/masked trials. The DMC is the only body involved in the trial that has access to unblinded/unmasked comparative data. The terms of reference and guidelines for DMCs are below.

Members should be completely independent of the CI, SSC, sponsor, funder and Host Institution.

#### Trial Oversight and Management

The role of the SSC is to provide overall supervision of the trial and ensure that the trial is conducted to the standards set out in these MRC Guidelines for Good Clinical Practice. In particular, the SSC should concentrate on progress of the trial, adherence to the approved protocol, patient safety and the consideration of new information. Day-to-day **management** of the trial is the responsibility of

the Chief Investigator and Principal Investigators at the sites. The Chief Investigators may wish to set up a separate Trial Management Group to assist with this function.

#### Patient Safety

In all the deliberations of the SSC the rights, safety and well-being of the trial participants are the most important considerations and should prevail over the interests of science and society. The SSC should ensure that the protocol demands freely given informed consent from every trial participant. The SSC should look closely at the patient information provided and advise the investigators on its completeness and suitability.

#### Progress of the Trial

It is the role of the SSC to monitor the progress of the trial and to maximise the chances of completing it within the time scale agreed by the MRC. At the first SSC meeting, targets for recruitment, data collection, compliance, etc. should be agreed with the Chief Investigator. These targets should not be "set in stone" but are designed to permit adequate monitoring of trial progress. The SSC should agree which data, based on the targets set, should be presented at each SSC meeting. The CI is required to submit an annual report to MRC based on the template provided. This report should be endorsed by the SSC and should be **stand alone** and contain sufficient data to allow MRC to judge progress in a trial without the need to refer back to the original grant proposal, and inform the MRC of any new information that has a bearing on safety or ethical acceptability of the trial or any significant complaints arising, with a justification of any decisions taken on the

matter.

In exceptional circumstances, Council will consider proposals for the extension of grants for clinical trials. In these cases, the MRC will require evidence from SSCs that all practicable steps have been taken to achieve targets and keep within the agreed duration of the grant. In these cases an analysis of the data collected to date that does not unblind/unmask the trial may be requested. If progress of the trial suggests that an extension may be necessary, the SSC should notify MRC officials at the earliest opportunity.

#### Adherence to Protocol

The full protocol should be presented as an agenda item at the first SSC meeting. The SSC should ensure that there are no major deviations from the trial protocol. If the Investigators recommend any material changes to the protocol during the course of the trial, approval should be sought from the SSC, the REC, and any appropriate regulatory body and the MRC should be informed.

#### Consideration of New Information

The SSC should consider new information relevant to the trial including reports from the DMC and the results of other studies. It is the responsibility of the CI the SSC Chair and other independent members of the SSC to bring to the attention of the SSC any results from other studies of which they are aware that may have a direct bearing on future conduct of the trial. On consideration of this information, the SSC should recommend appropriate action, such as changes to the trial protocol, additional patient information, or stopping or extending the study. The rights, safety and well-being of the trial participants (present and future) should be the most important considerations in

these deliberations. It is the responsibility of the investigators to report regularly the extent of

serious adverse events (SAEs) to the SSC and DMC. In case of unexpected SAEs the Chair of the SSC should be notified and, where appropriate the regulatory authority.

## Data Monitoring Committee (DMC)

### Terms of reference

1. to determine if additional interim analyses of trial data should be undertaken
2. to consider the data from interim analyses, unblinded if considered appropriate, plus any additional safety issues for the Roo*Pf*s study and relevant information from other sources
3. in the light of 2., and ensuring that the safety, rights and well being of the study participants are paramount, to report (following each DMC meeting) to the Study Steering Committee and to recommend on the continuation of the trial
4. to consider any requests for release of interim trial data and to recommend to the SSC on the advisability of this
5. in the event of further funding being required, to provide to the SSC and MRC appropriate information and advice on the data gathered to date that will not jeopardise the integrity of the study.

### Membership

The membership should be limited and include an independent chairman (Dr Immo Kleinschmidt), not less than two other independent members and one or two Principal Investigators. Trial co-ordinators, statisticians etc should attend meetings as appropriate. These are:

#### Independent members

Chair: Prof Brian Faragher (Statistician, Liverpool School of Tropical Medicine)

Prof Hilton Whittle (Clinician, London Sch. of Hygiene & Tropical Medicine) hcwhittle@yahoo.co.uk

Dr John Bradley (Statistician/epidemiologist, London Sch. of Hygiene & Tropical Medicine) John.Bradley@lshtm.ac.uk

#### Host organisation representative

Prof Umberto D’Alessandro (Chair, Director, MRC Unit, The Gambia)

#### Chair of SSC (invited to first meeting)

#### Study members

Prof Steve Lindsay (Chief Investigator)

Dr Margaret Pinder (Principal investigator)

Dr David Jeffries (Statistician)

### Guidance notes

1. The DMC should meet at least annually, or more often as appropriate, and meetings should be timed so that reports can be fed into the Study Steering Committee (SSC) meetings. Meetings should be called for and organised by the Chief Investigator of the trial in association with the Chair of the DMC. Dates for DMC meetings should be agreed in advance and only altered with agreement of all members. All significant communications between the Chief Investigator and the DMC should be in writing, or if they have to be oral, they should be backed up by written records.
2. The trial statistician should prepare a comprehensive report for the DMC. This should be prepared and circulated well in advance of the meeting to allow DMC members time to study the data. Content of the report should be agreed in advance with the DMC Chair. The trial statistician may be invited by the Chair to attend part of the meeting to present the data; otherwise, no one involved with the trial or SSC should be present to see the unblinded data.
3. A confidential report should be made in writing by the Chair of the DMC providing advice to the Study Steering Committee (and CI) on whether the trial should continue or not or be altered. If the DMC recommends that the trial should be stopped at any point, the funding body should be notified. It will be the responsibility of the SSC to decide whether or not to act upon the information received from the DMC.
4. If at any stage an extension to the grant is needed the DMC may be requested by the MRC Board to provide information on the data gathered to date (from this and other studies) and advice on the likelihood that continuation of the trial will allow detection of an important effect. This should be done using methods that do not unblind the trial.
5. Before reporting on the results of the trial the DMC will consider not only the interim results as presented by the trial statistician, but also any major new information from other sources thought to be relevant to the trial. It follows that the DMC will not automatically follow pre-assigned statistical rules, although it will be guided by statistical considerations.
6. Information provided by the DMC is likely to fall into the following categories:
7. Information that might lead to the SSC stopping the trial prematurely in the event of a clear outcome, if this is deemed to be appropriate in the light of the accumulating data from the study, or on the basis of information available from other sources;
8. Information that might lead to the SSC modifying the design of the trial, if this is deemed to be appropriate in the light of the accumulating data from the study or on the basis of information available from other sources.
